# Numerical investigation of acoustic vaporization threshold of microdroplets

**DOI:** 10.1016/j.ultsonch.2020.105361

**Published:** 2020-10-21

**Authors:** Sukwon Park, Gihun Son

**Affiliations:** Department of Mechanical Engineering, Sogang University, Seoul, South Korea

**Keywords:** Acoustic, Bubble collapse, Bubble rebound, Droplet vaporization, Threshold

## Abstract

A numerical model is presented for the acoustic vaporization threshold of a dodecafluoropentane (or perfluoropentane) microdroplet. The model is based on the Rayleigh-Plesset equation and is improved by properly treating the supercritical state that occurs when a bubble collapses rapidly and by employing the van der Waals equation of state to consider the supercritical state. The present computations demonstrate that the microdroplet vaporization behavior depends intricately on bubble compressibility, liquid inertia and phase-change heat transfer under acoustic excitation conditions. We present acoustic pressure-frequency diagrams for bubble growth regimes and the ADV threshold conditions. The effects of acoustic parameters, fluid properties and the droplet radius on the ADV threshold are investigated.

## Highlights

• A numerical method is presented for the acoustic vaporization threshold of microdroplets by improving the Rayleigh-Plesset equation to properly treat the supercritical state occurring at rapid bubble collapse.
• The van der Waals equation of state is employed to more accurately consider the supercritical state instead of the ideal gas equation.
• The effective ADV threshold is observed to increase as the acoustic frequency increases and the droplet radius decreases, as experimentally observed in the previous studies.
• The present numerical predictions of ADV threshold comparable to the experimental data.

## Nomenclature

a,b: constants in the van der Waals equation
$A_{a}$: pressure amplitude of acoustic pulse
*c*: specific heat
$f_{a}$: frequency of acoustic pulse
*G*: phase-change mass flux
$i_{\mathrm{bd}}$: latent heat of vaporization
*p*: pressure
*r*: radial coordinate
*R*: radius
$R_{g}$: gas constant
*t*: time
*T*: temperature

**Greek symbols**
γ: specific heat ratio
λ: thermal conductivity
μ: viscosity
ρ: density
σ: surface tension coefficient
ξ: moving coordinate, $r-R_{b}$

**Subscripts**
*b*: bubble
*c*: critical
*d*: droplet
*dth*: direct ADV threshold
*eth*: effective ADV threshold
*f*: final state at complete vaporization
*l*: liquid
*o*: initial
*sat*: saturation
*w*: ambient liquid water
∞: ambient

## 1 Introduction

Acoustic vaporization of volatile submicron droplets has received attention due to its medical potentials [1]. Due to the submicron size and stability of droplets, they can reach cancerous tissues through the endothelial cell wall [2] and vaporize into microbubbles that can be used as ultrasound contrast agents. The acoustic droplet vaporization (ADV) enables the targeted drug delivery and precise ultrasound imaging [3]. The large volume change and strong flow due to vaporization can be used for various medical therapies such as a gas embolotherapy and thermal ablation [4]. Despite of these potentials for medical applications, the use of ADV in the human body is challenging. The ADV requires a strong acoustic pulse beyond the critical amplitude, called the ADV threshold, to vaporize droplets, which can induce negative biological effects [5], [6].

Many experimental studies have been conducted for clarifying the ADV mechanism. Based on the previous experimental data for the acoustic threshold, Aliabouzer et al. [7] observed that the ADV threshold pressure decreases as the droplet size, ambient temperature, and number of acoustic cycles increase [8], [9], [10]. However, the effect of acoustic frequency on the ADV threshold was not consistent in the literature. Some studies showed that the ADV threshold increases with acoustic frequency [11], [12] whereas others showed the opposite trend [13], [14]. While analyzing the experimental observation that the acoustic threshold decreases as the acoustic frequency increases, Shpak et al. [15] noted the superharmonic effect that a spherical droplet concentrates an incident acoustic wave at its inner point. Their theoretical analysis showed that the superharmonic focusing effect, triggering the generation of bubbles, increases as the acoustic frequency and the droplet size increase. However, the superharmonic effect was observed to disappear in small droplets of 2 μm or less.

The ADV thresholds reported in the literature differ among researchers. It can be attributed not only to differences in threshold measurement techniques, such as hydrophone and imaging-based methods, but also to differences in experimental setups containing droplets, such as gel phantoms and tubes. The tube setups can cause uncertainties in threshold measurements through the scattering and reflection of acoustic waves. Aliabouzer et al. [7], [16] conducted experiments for the ADV threshold of microdroplets in a tubeless setup to minimize the effects of tube wall. Their results showed that the ADV threshold for dodecafluoropentane (DDFP) droplets increases with the acoustic frequency.

Theoretical studies have also been carried out for predicting the ADV threshold. Vlaisavljevich et al. [12] applied classical nucleation theory to prediction of the ADV threshold for perfluorohexane (PFH) nanodroplets used in histrotripsy. Miles et al. [17] extended the classical nucleation theory to the ADV threshold for DDFP microdroplets by combining with the superharmonic effect. Their predicted threshold pressure is about 9.3 MPa for a droplet of 10 μm radius, which is much higher than the experimental data of Aliabouzer et al. [16]. As another predictive model for the ADV threshold, one-dimensional numerical models for bubble growth have been applied to determine the conditions for the survival of a small bubble assumed to nucleate. The Rayleigh-Plesset (RP) equation for spherical bubble growth was extended for ADV by Qamar et al. [18] considering a tube geometry, Shpak et al. [19] including the effect of thermal boundary layer, Pitt et al. [20] investigating the effects of acoustic parameters, Doinikov et al. [21] computing the vapor generation rate from the energy equation, and Guédra and Coulouvrat [22] and Lacour et al. [23] including the effect of droplet encapsulation. The RP based numerical model has the advantage over the multidimensional numerical models for ADV developed in Refs. [24], [25], [26] that it can use a very fine mesh needed for the calculation of the initial bubble nucleus. Recently, Lacour et al. [27] applied the RP based numerical model to investigate the ultimate fate of a DDFP bubble nucleus forming in a DDFP droplet and determine the ADV threshold in various acoustic frequencies. The bubble growth pattern is observed to have three regimes: irreversible collapse without growth (regime I), direct vaporization without rebound (III) and intermediate behavior with collapses, rebounds and oscillations (regime II). They provided a phase diagram for the three regimes of bubble growth in ADV, which is useful to understand the complex bubble/droplet behaviors in ADV. However, their model needs to be modified in that the bubble has a very high temperature, to the order of a few MKs, while it collapses with oscillations in the intermediate regime III, and the ADV threshold is much higher than the experimental data of Aliabouzer et al. [16].

In this work, the RP based numerical model for predicting the ADV threshold is improved by properly treating the supercritical state occurring at rapid bubble collapse. The van der Waals (VDW) equation is employed to more accurately consider the supercritical state instead of the ideal gas equation, which was used in most of the previous studies referenced above for ADV. The present model is tested on the previous experimental data obtained in a tubeless setup [16]. The effects of fluid properties and droplet radius on the bubble growth pattern and the ADV threshold are investigated.

## 2 Mathematical modeling

**Fig. 1:**
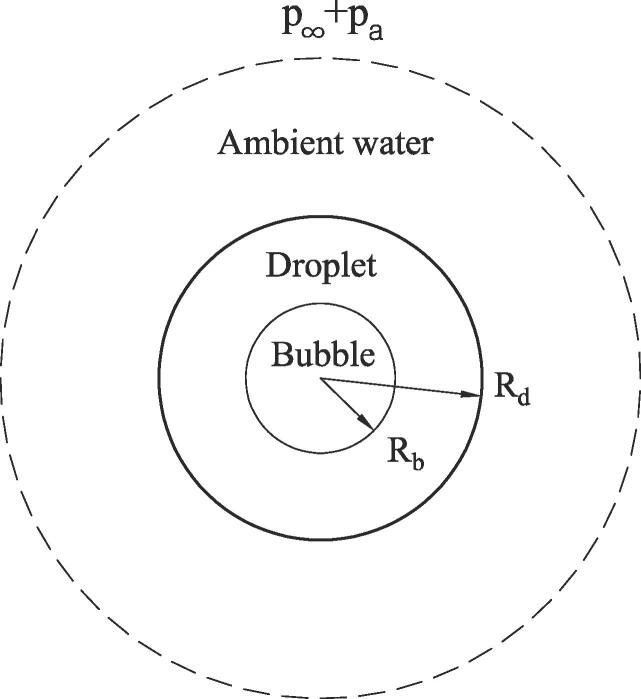
Schematic for modeling of ADV.

Fig. 1 shows the one-dimensional (spherical) schematic of ADV used in the present work. A spherical DDFP droplet with $R_{d}$ is immersed in ambient water, and a vapor bubble with $R_{b}$ is assumed to nucleate at the center of the droplet when the acoustic pressure pulse $p_{a}$ is excited from the boundary of the domain. The DDFP droplet and ambient water are considered incompressible fluids, and the DDFP bubble is considered a VDW gas rather than an ideal gas to more accurately consider the supercritical state occurring at bubble collapse. The VDW equation of state is expressed as

(1) $$p_{b}=\frac{\rho_{b}R_{g}T_{b}}{1-b\rho_{b}}-a\rho_{b}^{2}$$

where $R_{g}$ is the gas constant and the constants *a* and *b* are evaluated as [28]

(2) $$a=\frac{27}{64}\frac{R_{g}^{2}T_{c}^{2}}{p_{c}},b=\frac{R_{g}T_{c}}{8p_{c}}$$

Here, the subscript c denotes the critical state.

### 2.1 Governing equations

Assuming that the vapor pressure, density and temperature are uniform, the one-dimensional conservation equations in the liquid (droplet and ambient water) regions as well as the mass equation in the bubble region are expressed as

(3) $$\frac{1}{r^{2}}\frac{\partial }{\partial r}(r^{2}u_{b})=-\frac{1}{\rho_{b}}\frac{\partial \rho_{b}}{\partial t}\;\mathrm{if}\;r<R_{b}$$

(4) $$\frac{1}{r^{2}}\frac{\partial }{\partial r}(r^{2}u_{l})=0\;\mathrm{if}\;r>R_{b}$$

(5) $$\rho_{l}(\frac{\partial u_{l}}{\partial t}+u_{l}\frac{\partial u_{l}}{\partial r})=-\frac{\partial p_{l}}{\partial r}\;\mathrm{if}\;r>R_{b}$$

(6) $${\left(\rho c\right)}_{l}\left(\frac{\partial T_{l}}{\partial t}+u_{l}\frac{\partial T_{l}}{\partial r}\right)=\frac{1}{r^{2}}\frac{\partial }{\partial r}\left(r^{2}\lambda_{l}\frac{\partial T_{l}}{\partial r}\right)\;\;+2\mu_{l}\left[{\left(\frac{\partial u_{l}}{\partial r}\right)}^{2}+2{\left(\frac{u_{l}}{r}\right)}^{2}\right]\;\;\mathrm{if}\;\;r>R_{b}$$

where the subscripts b and l indicate bubble and liquid regions, respectively. The conservation equations are solved using the matching conditions at the bubble surface ($r=R_{b}$) with phase change,

(7) $$\rho_{b}(\overset{˙}{R_{b}}-U_{b})=\rho_{d}(\overset{˙}{R_{b}}-U_{d})=G$$

(8) $$p_{b}-p_{d}=\frac{2\sigma_{\mathrm{bd}}}{r}-(\frac{1}{\rho_{b}}-\frac{1}{\rho_{d}})G^{2}+2\mu_{b}\frac{\partial u_{b}}{\partial r}-\frac{2}{3}\frac{\mu_{b}}{r^{2}}\frac{\partial r^{2}u_{b}}{\partial r}-2\mu_{d}\frac{\partial u_{d}}{\partial r}$$

(9) $$T_{d}=T_{b}$$

(10) $$G=\frac{1}{i_{\mathrm{bd}}}\lambda_{d}\frac{\partial T_{d}}{\partial r}$$

and the matching conditions at the droplet surface ($r=R_{d}$) without phase change,

(11) $$u_{d}=u_{w}$$

(12) $$p_{d}-p_{w}=\frac{2\sigma_{\mathrm{dw}}}{r}+2\mu_{d}\frac{\partial u_{d}}{\partial r}-2\mu_{w}\frac{\partial u_{w}}{\partial r}$$

(13) $$T_{d}=T_{w}$$

(14) $$\lambda_{d}\frac{\partial T_{d}}{\partial r}=\lambda_{w}\frac{\partial T_{w}}{\partial r}$$

Here, the subscripts d and w denote droplet and ambient water regions, respectively, *G* is the phase-change mass flux, $\overset{˙}{R_{b}}$ is the bubble surface velocity, and $U_{b}$ and $U_{d}$ are the vapor and liquid velocities at $r=R_{b}$, respectively. The boundary condition at r=∞ is specified as

(15) $$u_{w}=0$$

(16) $$p_{w}=p_{\infty }+p_{a}$$

(17) $$T_{w}=T_{\infty }$$

where $p_{\infty }$ and $p_{a}$ are ambient and acoustic pressures, respectively.

The vapor and liquid velocity profiles are solved from Eqs. (3), (4) with the boundary conditions as

(18) $$u_{b}=U_{b}\frac{r}{R_{b}},\;u_{l}=U_{d}\frac{R_{b}^{2}}{r^{2}}$$

Assuming $\rho_{d}\gg \rho_{b}$, integration of the momentum equations over the droplet and ambient water regions with the boundary conditions result in the Rayleigh-Plesset equation [21], [26],

(19) $$\rho_{d1}R_{b}\frac{{\mathrm{dU}}_{d}}{\mathrm{dt}}=-2\rho_{d1}\overset{˙}{R_{b}}U_{d}+\frac{1}{2}\rho_{d2}U_{d}^{2}+p_{b}-p_{\infty }-p_{a}+\left(\frac{1}{\rho_{b}}-\frac{1}{\rho_{d}}\right)G^{2}-\frac{2\sigma_{\mathrm{bd}}}{R_{b}}-\frac{2\sigma_{\mathrm{dw}}}{R_{d}}-4\mu_{d1}\frac{U_{d}}{R_{b}}$$

where

(20) $$\rho_{d1}=\rho_{d}+(\rho_{w}-\rho_{d})\frac{R_{b}}{R_{d}}$$

(21) $$\rho_{d2}=\rho_{d}+(\rho_{w}-\rho_{d})\frac{R_{b}^{4}}{R_{d}^{4}}$$

(22) $$\mu_{d1}=\mu_{d}+(\mu_{w}-\mu_{d})\frac{R_{b}^{3}}{R_{d}^{3}}$$

A fourth-order Runge–Kutta (RK) method with an adaptive computational time step is used to solve $U_{d}$ from Eq. (19), which includes *G*, whose calculation procedure will be described later, $R_{b}$ to be determined from Eq. (7), $\rho_{b}$ from Eq. (23) for the bubble mass conservation, $R_{d}$ from Eq. (24) for the droplet mass conservation, $p_{b}$ and $T_{b}$ from the VDW Eq. (1) combined with the Clausius–Clapeyron Eq. (25).

(23) $$\frac{d}{\mathrm{dt}}(\rho_{b}R_{b}^{3})=3R_{b}^{2}G\;\;\;\mathrm{or}\;\;\;\frac{d\rho_{b}}{\mathrm{dt}}=\frac{3}{R_{b}}(G-\rho_{b}{\overset{˙}{R}}_{b})$$

(24) $$\frac{d}{\mathrm{dt}}[\rho_{d}(R_{d}^{3}-R_{b}^{3})]=-3R_{b}^{2}G\;\;\;\mathrm{or}\;\frac{{\mathrm{dR}}_{d}}{\mathrm{dt}}=-\frac{R_{b}^{2}}{R_{d}^{2}}\left(\frac{G}{\rho_{d}}-{\overset{˙}{R}}_{b}\right)$$

(25) $$p_{b}=p_{\infty }\exp\left[\frac{i_{\mathrm{bd}}}{R_{g}}\left(\frac{1}{T_{b,\infty }}-\frac{1}{T_{b}}\right)\right]$$

where $T_{b,\infty }$ is the saturation temperature at $p_{\infty }$. It is noted that Eq. (23) has an advantage in preserving the bubble mass over Eq. (26) used in many previous studies [21], [22], [23], [27], which can be derived from Eq. (23) and the thermodynamic relation $\left(d\rho_{b}/\mathrm{dt})=(\rho_{b}/\gamma_{b}p_{b})({\mathrm{dp}}_{b}/\mathrm{dt}\right)$ in the isentropic process.

(26) $$\frac{{\mathrm{dp}}_{b}}{\mathrm{dt}}=\frac{3\gamma_{b}p_{b}}{\rho_{b}R_{b}}(G-\rho_{b}{\overset{˙}{R}}_{b})$$

To calculate the phase-change mass flux *G* from Eq. (10), the liquid-side energy Eq. (6) is solved with the boundary condition of $T=T_{b}$ at the moving bubble surface. Introducing the moving coordinate $\xi =r-R_{b}$, Eq. (6) is rewritten as

(27) $${\left(\rho c\right)}_{l}\left[\frac{\partial T_{l}}{\partial t}+\left(\frac{R_{b}^{2}}{r^{2}}U_{d}-\overset{˙}{R_{b}}\right)\frac{\partial T_{l}}{\partial \xi }\right]=\frac{1}{r^{2}}\frac{\partial }{\partial \xi }\left(r^{2}\lambda \frac{\partial T_{l}}{\partial \xi }\right)\;\;+\frac{12\mu_{l}U_{d}^{4}}{r^{6}}$$

where the subscript l represents the droplet or ambient water region when the computational cell is a single-phase cell that does not include the droplet surface ($r=R_{d}$). For a droplet-water mixture cell, ${\left(\rho c\right)}_{l},\mu_{l}$ and $\lambda_{l}$ are interpolated from the droplet and water properties, as done in Ref. [25] using a level-set method. Eq. (27) is spatially discretized using second-order essentially non-oscillatory (ENO) and central difference schemes for the convection and diffusion terms, respectively. A fourth-order RK method with an adaptive time step is applied to solve the transient differential equation.

While a bubble collapses rapidly in ADV, its pressure (or temperature) can exceed a critical value. To simulate the supercritical process ($p_{b}⩾p_{c}$ or $T_{b}⩾T_{c}$), where liquid and vapor phases are not distinguished, we assume that the phase-change mass flux is zero (G=0), as done by Akhatov et al. [29] for computing a laser-induced bubble, and the bubble state varies in an isentropic process, which is expressed as [28]

(28) $$\frac{c_{v}}{T}\mathrm{dT}=-{\left(\frac{\partial p}{\partial T}\right)}_{\rho }d\left(\frac{1}{\rho }\right)$$

Integration of Eq. (28) using the VDW Eq. (1) yields

(29) $$T_{b}=T_{c}{\left(\frac{\rho_{c}^{-1}-b}{\rho_{b}^{-1}-b}\right)}^{\gamma_{b}-1}$$

The bubble temperature is determined from Eq. (29) if $\rho_{b}⩾\rho_{c}$ and from the combined Eqs. (1), (25) if $\rho_{b}<\rho_{c}$.

### 2.2 Computational conditions

We consider acoustic vaporization of a DDFP droplet in water at $p_{\infty }$ =1 atm and $T_{\infty }=310\;\text{K}$, as depicted in Fig. 1. The DDFP and water properties are chosen from Refs. [21], [23], [30] as $\rho_{d}=1.63\times 10^{3}\;{\text{kg/m}}^{3},\;\mu_{d}=6.52\times 10^{-4}\;\text{Pas}$, $c_{d}=1.09\times 10^{3}\;\text{J/kgK},\;\lambda_{d}=5.6\times 10^{-2}\;\text{W/mK}$, $R_{\mathrm{gb}}=28.8\;\text{J/kgK},\;\gamma_{b}=1.05,\;\sigma_{\mathrm{bd}}=9.50\times 10^{-3}\;\text{N/m}$, $i_{\mathrm{bd}}=8.8\times 10^{4}\;\text{J/kg},\;T_{b,\infty }=302\;\text{K}$, $\rho_{w}=9.98\times 10^{2}\;{\text{kg/m}}^{3},\;\mu_{w}=1\times 10^{-3}\;\text{Pas}$, $c_{w}=4.2\times 10^{3}\;\text{J/kgK},\;\lambda_{w}=0.6\;\text{W/mK}$, $\sigma_{\mathrm{dw}}=5\times 10^{-2}\;\text{N/m}$ and $p_{c}=2.045\;\text{MPa}$. The bubble density is assumed to follow the van der Waals Eq. (1).

For computation of ADV, we use the following acoustic pressure pulse.

(30) $$p_{a}=-A_{a}\sin(2\pi f_{a}t)\;\mathrm{if}\;0⩽t⩽N_{a}/f_{a}$$

$$=0\;\;\;\;\mathrm{if}\;t⩾N_{a}/f_{a}$$

Here, $A_{a}$ and $f_{a}$ vary in this work keeping the number of acoustic cycles as $N_{a}=8$, referring to the experimental condition of Aliabouzer et al. [16]. The minus sign is chosen considering that bubble nucleation occurs in the negative pressure period, as experimentally observed in Ref. [31].

As the initial conditions, we choose $R_{\mathrm{do}}=0.47\;\mu \text{m}$ as the droplet radius and $T_{l}=T_{\infty }=310\;\text{K}$ as the liquid temperature profile, based on Ref. [16]. The initial bubble radius $R_{\mathrm{bo}}$ is unknown and can be considered an external parameter. In this work, we choose $R_{\mathrm{bo}}=80\;\text{nm}$ as a base case, which was also used by Lacour et al. [23], [27] while predicting the ADV threshold of DDFP droplets.

Introducing the time $t_{\mathrm{bo}}$ at bubble nucleation after the acoustic pulsing starts and considering the Laplace pressure caused by the surface tensions at $r=R_{\mathrm{bo}}$ and $r=R_{\mathrm{do}}$, the initial bubble pressure $p_{\mathrm{bo}}$ can be estimated as

(31) $$p_{\mathrm{bo}}=\frac{2\sigma_{\mathrm{bd}}}{R_{\mathrm{bo}}}+\frac{2\sigma_{\mathrm{dw}}}{R_{\mathrm{do}}}+p_{\infty }-A_{a}\sin(2\pi f_{a}t_{\mathrm{bo}})$$

The bubble nucleation time $t_{\mathrm{bo}}$ is another parameter affecting the bubble growth in ADV. In the case of $t_{\mathrm{bo}}=0$, which means that bubble nucleation occurs when the acoustic pulsing starts, we obtain $p_{\mathrm{bo}}=0.552\;\text{MPa}$ from Eq. (31) and the corresponding bubble temperature of $T_{\mathrm{bo}}=363\;\text{K}$ from Eq. (25). This case has a mismatch of $T_{l}\;\neq \;T_{b}$ near the bubble surface ($r=R_{b}$) and thus the initial droplet temperature profile needs to be modified as done in the previous studies [19], [22] introducing a thin liquid layer for smooth transition from $T_{b}$ to $T_{\infty }$.

As another case of $t_{\mathrm{bo}}$, we assume that a bubble nucleates when its nucleus is in thermal equilibrium with the surrounding liquid at $T_{\infty }$. Assuming $T_{\mathrm{bo}}=T_{\infty }=310\;\text{K}$, the corresponding bubble pressure is estimated as $p_{\mathrm{bo}}=0.131\;\text{MPa}$ from Eq. (25). The acoustic amplitude required for bubble nucleation is $A_{a}>0.42\;\text{MPa}$ and given $A_{a}$, the bubble generation time $t_{\mathrm{bo}}$ is determined from Eq. (31).

The liquid-side energy Eq. (27) formulated in a moving coordinate is solved in a computational domain of 0<ξ<L (or $R_{b}<r<L-R_{b}$). We use uniform fine meshes of size Δr in a region of $0<\xi <15R_{\mathrm{do}}$ and nonuniform meshes in the outer region of $15R_{\mathrm{do}}<\xi <10^{6}R_{\mathrm{do}}$.

**Fig. 2:**
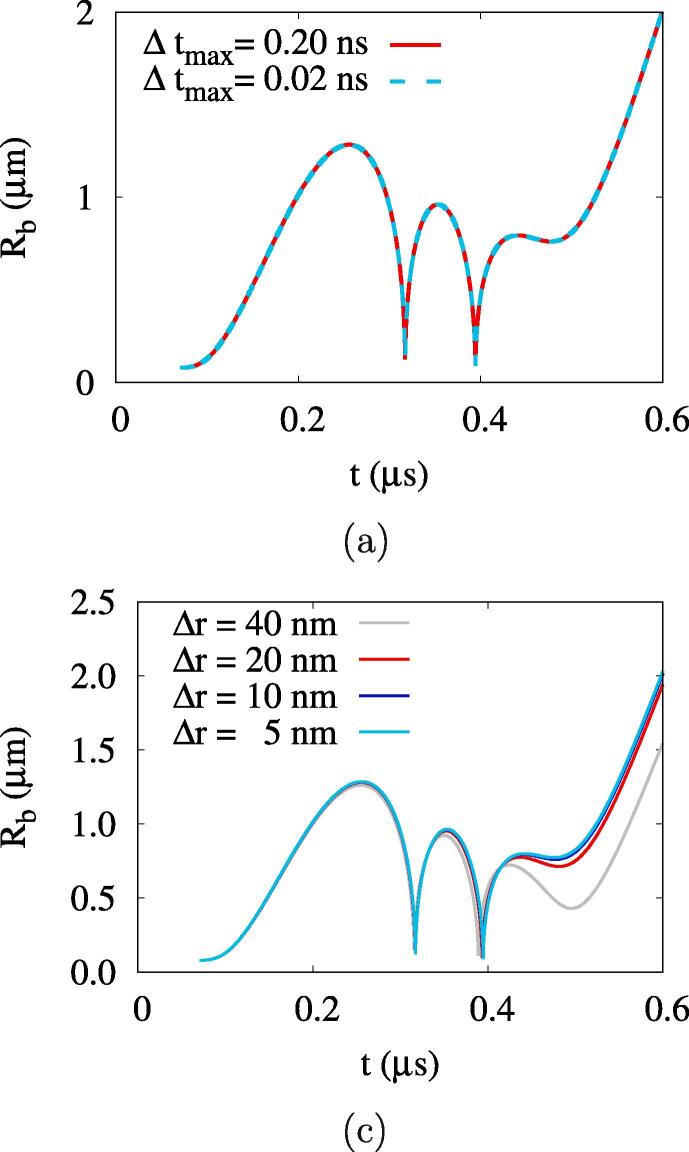
Effects of (a) time step and (b) mesh size on the computed bubble growth for ADV at $R_{\mathrm{do}}=0.47\;\mu \text{m},A_{a}=0.50\;\text{MPa}$ and $f_{a}=2.25\;\text{MHz}$.

Time and space resolutions are tested to determine the proper time step Δt and mesh size Δr, as plotted in Fig. 2. The bubble growth computed with different time steps has no difference while using adaptive time steps with a maximum of Δt⩽0.2ns. In Fig. 2b, the results obtained with different mesh sizes show that the bubble oscillations converge as the mesh size decreases below Δr=10nm. Therefore, we use Δr=10nm and the adaptive time step in the present computations.

## 3 Results and discussion

### 3.1 ADV at $R_{\mathrm{do}}=0.47\;\mu \text{m}$ and $f_{a}=2.25\;\text{MHz}$

**Fig. 3:**
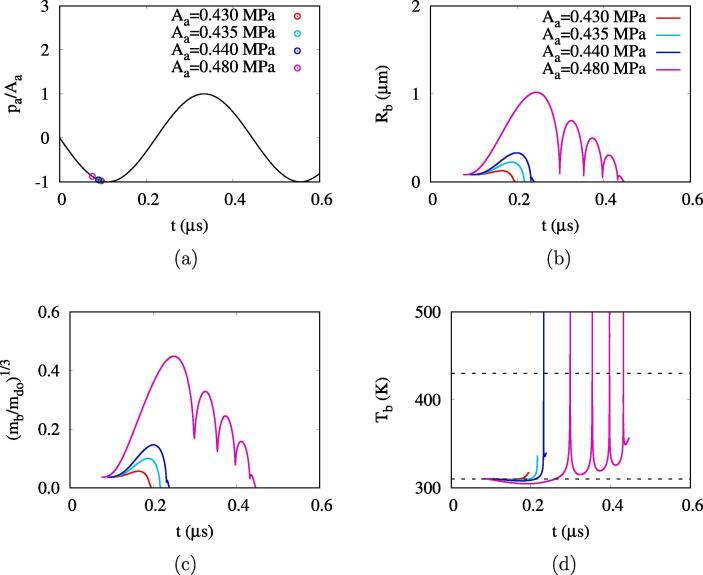
ADV at $R_{\mathrm{do}}=0.47\;\mu \text{m},f_{a}=2.25\;\text{MHz}$ and $0.43\;\text{MPa}⩽A_{a}⩽0.48\;\text{MPa}$: (a) acoustic pressure and bubble nucleation time (marked by circle symbols), (b) bubble radius, (c) bubble mass and (d) bubble temperature. In the figure d, the dashed lines represent $T_{\infty }=310\;\text{K}$ and $T_{c}=430\;\text{K}$.

**Fig. 4:**
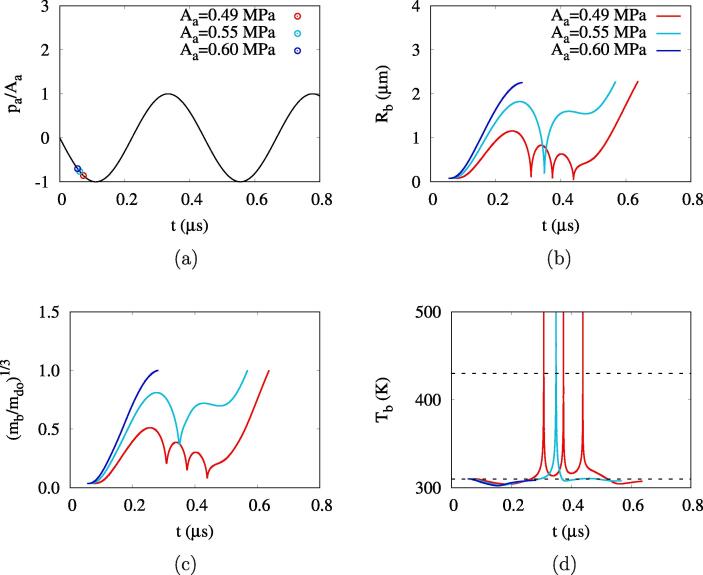
ADV at $R_{\mathrm{do}}=0.47\;\mu \text{m},f_{a}=2.25\;\text{MHz}$ and $0.49\;\text{MPa}⩽A_{a}⩽0.60\;\text{MPa}$: (a) acoustic pressure and bubble nucleation time (marked by circle symbol), (b) bubble radius, (c) bubble mass and (d) bubble temperature. In the figure d, the dashed lines represent $T_{\infty }=310\;\text{K}$ and $T_{c}=430\;\text{K}$.

Fig. 3, Fig. 4 show the results for ADV in a base case of $R_{\mathrm{do}}=0.47\;\mu \text{m},f_{a}=2.25\;\text{MHz}$ and different acoustic amplitudes of $0.4\;\text{MPa}\;⩽A_{a}⩽0.6\;\text{MPa}\;$. For $A_{a}=0.43\;\text{MPa}\;$, a bubble is assumed to nucleate at $t_{\mathrm{bo}}=0.096\;\mu \text{s}$, as marked by circle symbols in Fig. 3a, when the bubble temperature is reduced to $T_{\infty }=310\;\text{K}$ by the acoustic pressure. The bubble grows during a low pressure pulsing period of $t_{\mathrm{bo}}<t<0.165\;\mu \text{s}$ and reaches its maximum radius of $R_{b}=0.13\;\mu \text{m}$. As the bubble pressure $p_{b}$ decreases with bubble growth and the corresponding $T_{b}$ decreases below $T_{\infty }$, the bubble mass $m_{b}$ increases, as seen in Fig. 3c, because of the evaporative heat transfer from the droplet bulk. Thereafter, while the acoustic pressure increases, the bubble shrinks rapidly and $p_{b}$ increases. As $T_{b}$ becomes higher than $T_{\infty },m_{b}$ decreases because of to the condensing heat transfer from the bubble surface. The computation is carried out until the bubble and its mass become very small to $R_{b}<0.01R_{\mathrm{bo}}$ and $m_{b}^{1/3}<0.01m_{\mathrm{bo}}^{1/3}$. This result means that the bubble nucleus collapses with no rebound at $A_{a}=0.43\;\text{MPa}$. The irreversible bubble collapse occurs in the acoustic amplitude range of $A_{a}⩽0.435\;\text{MPa}$, which is referred to bubble growth regime 1 in the present work. In this regime, the droplet size hardly changes with acoustic pulsing, which was also observed in the previous experimental studies [8], [32] using high speed microscopy to investigate the ADV behavior.

When the acoustic amplitude increases to $0.44\;\text{MPa}⩽A_{a}⩽0.48\;\text{MPa}$, a bubble nucleates earlier and grows larger during the first negative pulsing period ($t<0.5/f_{a}=0.22\;\mu \text{s}$). However, while the positive pulsing is applied (0.22μs<t<0.44μs), the bubble collapses and rebounds, which is caused by the unbalance between liquid inertia and bubble pressure. The number of bubble collapse and rebound increases with $A_{a}$. In Fig. 3d, while the bubble collapses due to the inertia of the surrounding droplet and ambient water, its temperature exceeds the critical temperature of $T_{c}=430\;\text{K}$, which is evaluated from Eq. (25) with $p_{c}=2.045\;\text{MPa}$ for DDFP. In this work, we assume G=0 at the supercritical state. As the bubble collapse builds up the bubble pressure and also the liquid pressure, the bubble rebounds rapidly. During the positive pulsing period, the bubble at the subcritical state ($T_{b}<T_{c}$) has a higher temperature than $T_{\infty }$ and thus $m_{b}$ decreases to zero. The acoustic amplitude range of $0.44\;\text{MPa}⩽A_{a}⩽0.48\;\text{MPa}$, where the irreversible bubble collapse occurs after multiple collapses and rebounds, is referred to bubble growth regime 2. This regime corresponds to the experimental observation of Sheeran et al. [32] that some droplets expand during the negative cycle of acoustic pulsing and then disappear during the positive pulsing period.

When the acoustic amplitude further increases to $0.49\;\text{MPa}⩽A_{a}⩽0.59\;\text{MPa}$, the negative pulsing causes the bubble to grow enough to survive during the following positive pulsing period, as seen in Fig. 4b. It is noted that whenever the bubble collapses and rebounds, its temperature exceeds the critical temperature (Fig. 4d). and thus proper modeling of the supercritical process is essentially important for simulating bubble behavior after collapse and rebound. When the second negative pulsing period begins at t=0.44μs, the bubble grows again and then reaches its maximum radius of $R_{b,f}=2.26\;\mu \text{m}$ at the time of complete vaporization. The acoustic amplitude range of $0.49\;\text{MPa}⩽A_{a}⩽0.59\;\text{MPa}$, where the droplet vaporization is complete after bubble collapses and rebounds, is referred to bubble growth regime 3. This bubble growth behavior was observed in several experimental studies [8], [31], [32], [33], [34]. The minimum acoustic pressure required for complete droplet vaporization is $A_{a}=0.49\;\text{MPa}$. As $A_{a}$ increases above 0.6 MPa, the droplet vaporization is directly complete during the first negative pulsing period, which is called bubble growth regime 4. The minimum pressure for direct droplet vaporization without any collapse and rebound is called the direct threshold in Refs. [23], [27]. This bubble growth regime was observed by Li et al. [34] while visualizing the formation of toroidal bubbles from high acoustic pressure.

**Fig. 5:**
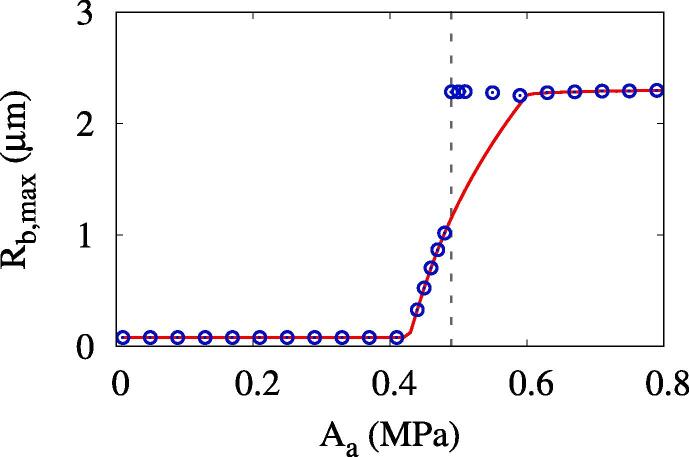
Maximum bubble radius for ADV at $R_{\mathrm{do}}=0.47\;\mu \text{m},f_{a}=2.25\;\text{MHz}$ and various acoustic amplitudes. In the figure, the red line and blue circle symbols represent the first local and global maxima in the vaporization period, respectively, and the vertical dashed line the effective ADV threshold. (For interpretation of the references to colour in this figure legend, the reader is referred to the web version of this article.)

Fig. 5 presents the first local maximum bubble radius $R_{b,\mathrm{lmax}}$, which is observed during the first negative pulsing period, and the global maximum bubble radius $R_{b,\mathrm{gmax}}$ in the vaporization period. It is seen that $R_{b,\mathrm{lmax}}$ increases linearly with $A_{a}$. The bubble growth regimes are distinguished as follows: regimes 1 and 2 if $R_{b,\mathrm{gmax}}<R_{b,f}$, regime 3 if $R_{b,\mathrm{lmax}}<R_{b,\mathrm{gmax}}=R_{b,f}$, and regime 4 if $R_{b,\mathrm{lmax}}=R_{b,\mathrm{gmax}}=R_{b,f}$. The effective ADV threshold $A_{\mathrm{eth}}$ is defined in this work as the minimum acoustic amplitude that satisfies $R_{b,\mathrm{gmax}}(A_{a}⩾A_{\mathrm{eth}})=R_{b,f}$. The effective ADV threshold is $A_{\mathrm{eth}}=0.49\;\text{MPa}$ and the direct ADV threshold is $A_{\mathrm{dth}}=0.60\;\text{MPa}$ in the base case.

### 3.2 Effect of frequency of acoustic pulse

**Fig. 6:**
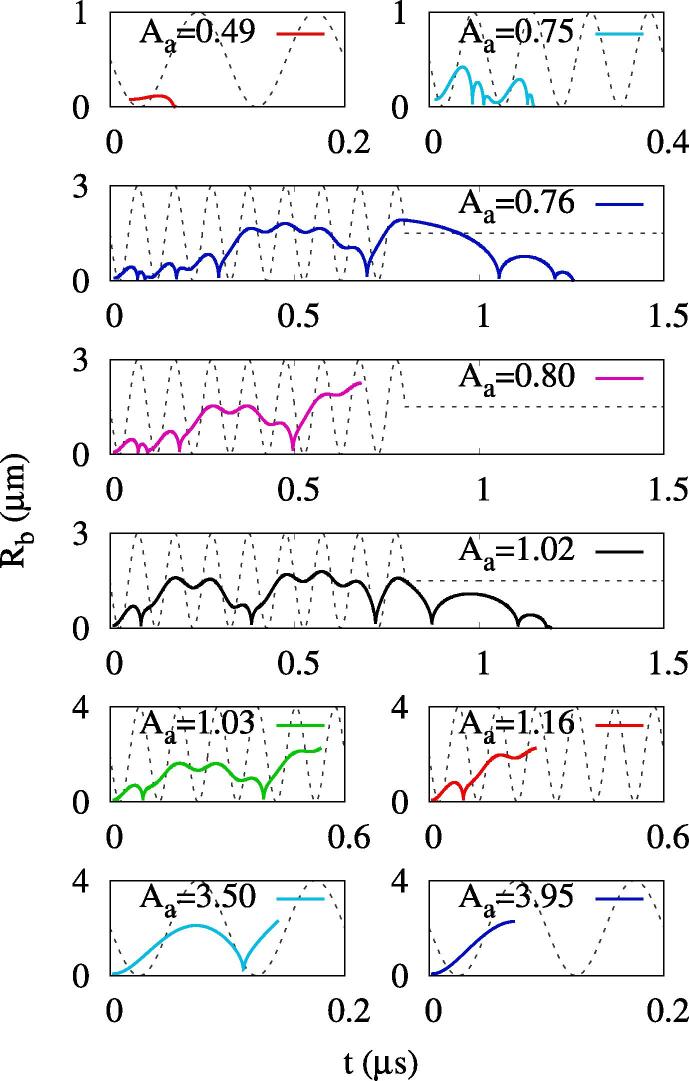
Bubble growth for ADV at $R_{\mathrm{do}}=0.47\;\mu \text{m}$ and $f_{a}=10\;\text{MHz}$. The dashed lines represents the acoustic pressure drawn on arbitrary scales and the acoustic amplitude $A_{a}$ in MPa.

Fig. 6 shows bubble growth at an acoustic frequency increased to $f_{a}=10\;\text{MHz}$. For $A_{a}=0.49\;\text{MPa}$, which is the effective ADV threshold at $f_{a}=2.25\;\text{MHz}$, a bubble immediately collapses without rebound because the first negative acoustic pulsing period is significantly reduced in this high frequency case. As $A_{a}$ increases, the maximum bubble radius increases and the bubble oscillations including collapses and rebounds become pronounced. For $A_{a}=0.76\;\text{MPa}$, a bubble grows with oscillations during the acoustic pulsing periods of $8/f_{a}$ and thereafter it shrinks with multiple collapses and rebounds and finally disappears.

**Fig. 7:**
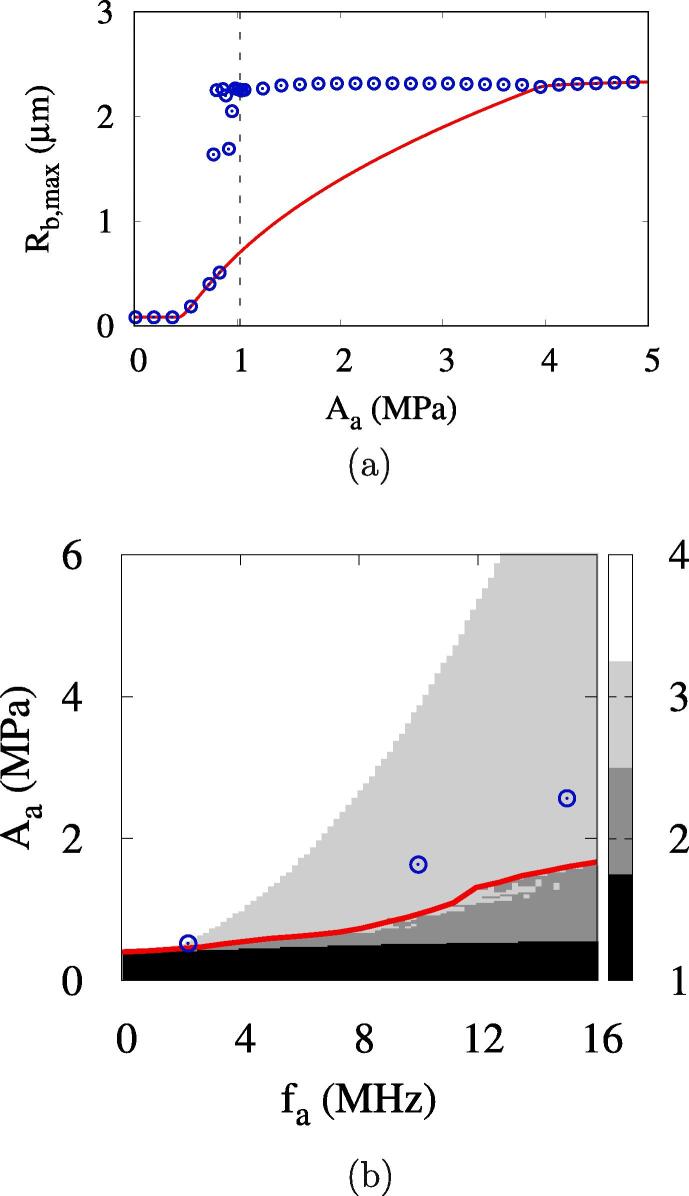
ADV at $R_{\mathrm{do}}=0.47\;\mu \text{m}$: (a) the maximum bubble radius at $f_{a}=10\;\text{MHz}$ and (b) the bubble growth regimes. In the figure a, the red line and blue circle symbols represent the first local and global maxima in the vaporization period, respectively, and the vertical dashed line the effective ADV threshold. In the figure b, the red lines and blue circles represent the present numerical predictions of the ADV threshold and the experiment data [16]., respectively. (For interpretation of the references to colour in this figure legend, the reader is referred to the web version of this article.)

The acoustic amplitude increased to $A_{a}=0.80\;\text{MPa}$ causes complete vaporization after multiple bubble oscillations, but a higher amplitude of $A_{a}=1.02\;\text{MPa}$ causes irreversible bubble collapse after multiple rebounds. This indicates that the ADV becomes more complicated under high frequency conditions and the bubble growth regimes 2 and 3 are not clearly distinguished by the acoustic amplitude, which was also observed by Lacour et al. [27]. The complete vaporization occurs in the acoustic amplitude range of $A_{a}⩾1.03\;\text{MPa}$, as depicted in Fig. 7a. The effective ADV threshold of $A_{\mathrm{eth}}=1.03\;\text{MPa}$ and the direct ADV threshold of $A_{\mathrm{dth}}=3.95\;\text{MPa}$ are much higher than those at $f_{a}=2.25\;\text{MHz}$.

Fig. 7b presents the acoustic pressure-frequency diagram for bubble growth regimes. In this work, the bubble growth regimes are divided into four regimes indicating (1) irreversible collapse with no rebound, (2) irreversible collapse after multiple rebounds, (3) complete vaporization after collapses and rebounds and (4) complete vaporization with no collapse. The acoustic pressure-frequency diagram for bubble growth regimes seems to be similar to that proposed by Lacour et al. [27], but there is a significant difference in regime 3 for complete vaporization after collapses and rebounds. Considering the acoustic amplitude range of $A_{\mathrm{eth}}⩽A_{a}⩽A_{\mathrm{dth}}$ at a high frequency of $f_{a}=10\mathrm{MHz}$, where bubble growth is large in the first negative pulsing period, as seen at $A_{a}=3.50\;\text{MPa}$ of Fig. 6, modeling of bubble collapse is expected to be very important for the subsequent bubble behavior. The present analysis of the rapid collapse of a large bubble, which occurs at the supercritical state (Fig. 4d), results in the rebound and complete vaporization of the bubble, whereas the analysis of Lacour et al. [27], extending the phase-change formulation to the supercritical state, resulted in the irreversible collapse of the bubble with a very high temperature of a few MKs (regime IIb of Ref. [27]). The experimental data for the ADV threshold of Aliabouzer et al. [16] are within complete vaporization regime 3 and are more comparable to the present prediction of $A_{\mathrm{eth}}$, indicated by the red line in Fig. 7b, than the direct ADV threshold $A_{\mathrm{dth}}$. This indicates that the present numerical model for ADV is improved by properly treating the supercritical state at bubble collapse.

### 3.3 Effects of bubble nucleation time and initial bubble radius

**Fig. 8:**
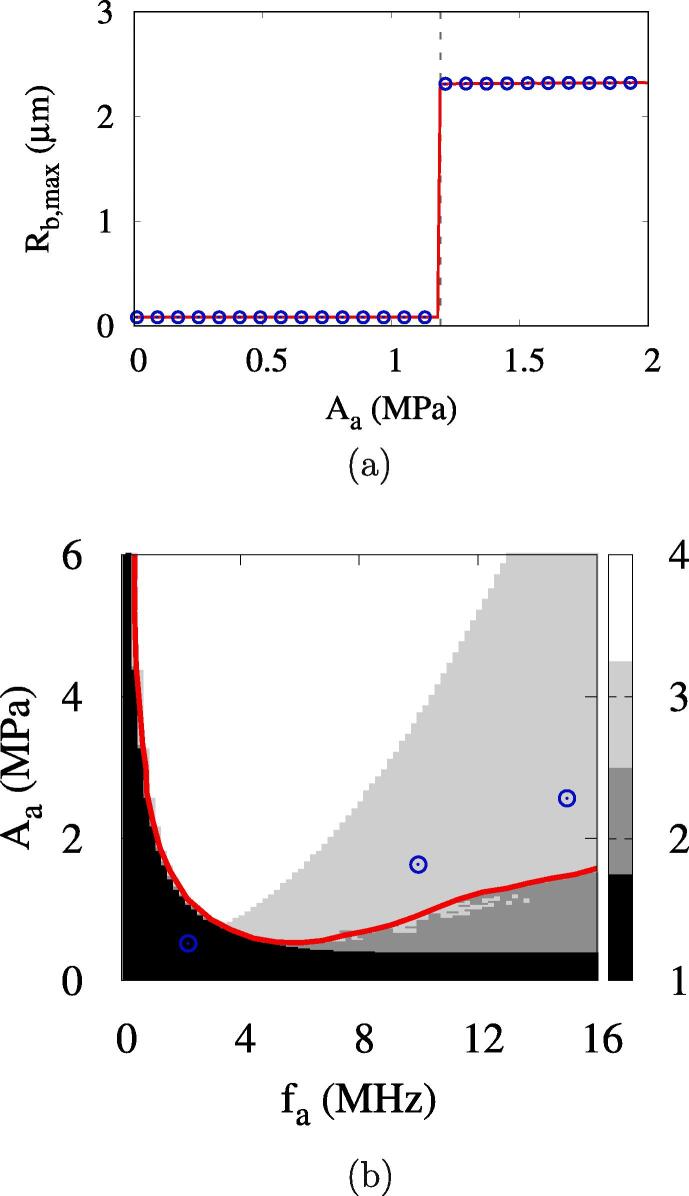
ADV with bubble generation time changed to $t_{\mathrm{bo}}=0$ at $R_{\mathrm{do}}=0.47$: (a) the maximum bubble radius at $f_{a}=2.25\;\text{MHz}$ and (b) the bubble growth regimes. In the figure a, the red line and blue circle symbols represent the first local and global maxima in the vaporization period, respectively, and the vertical dashed line the effective ADV threshold. In the figure b, the red lines and blue circles represent the present numerical predictions of the ADV threshold and the experiment data [16]., respectively. (For interpretation of the references to colour in this figure legend, the reader is referred to the web version of this article.)

Fig. 8 shows ADV at a bubble nucleation time changed to $t_{\mathrm{bo}}=0$, which was used in the previous studies [23], [27]. If a bubble nucleates at $t_{\mathrm{bo}}=0$ when the acoustic pulsing starts, its pressure is estimated from Eq. (31) including the Laplace pressure contribution as $p_{\mathrm{bo}}=0.552\;\text{MPa}$. The corresponding bubble temperature of $T_{\mathrm{bo}}=363\;\text{K}$ evaluated from Eq. (25) is higher than $T_{\infty }=310\;\text{K}$, and thus the nucleated bubble initially condenses until its temperature drops below $T_{\infty }$ by negative acoustic pulsing.

For a low frequency of $f_{a}=2.25\mathrm{MHz}$, the bubble growth regimes are sharply distinguished as depicted in Fig. 8a. The bubble collapses with no rebound in the acoustic amplitude range of $A_{a}⩽1.18\;\text{MPa}$, whereas the bubble directly vaporizes with no collapse for $A_{a}⩾1.19\;\text{MPa}$. The effective and direct ADV thresholds are the same as $A_{\mathrm{eth}}=A_{\mathrm{dth}}=1.19\;\text{MPa}$, which is much higher than $p_{\mathrm{eth}}=0.49\;\text{MPa}$ for ADV at the bubble nucleation time determined in Section 3.1.

The acoustic pressure-frequency diagram for bubble growth regimes is plotted in Fig. 8b. Compared with Fig. 7b, the bubble growth regimes are quite different at low frequencies although the difference becomes small at high frequencies. The prediction of $A_{\mathrm{eth}}$ with $t_{\mathrm{bo}}=0$ is deviated from the experimental data of Aliabouzer et al. [16] for ADV at a low frequency. This indicates that the present assumption of bubble nucleation when the bubble nucleus is in thermal equilibrium with the surrounding liquid is reasonable for analysis of ADV.

**Fig. 9:**
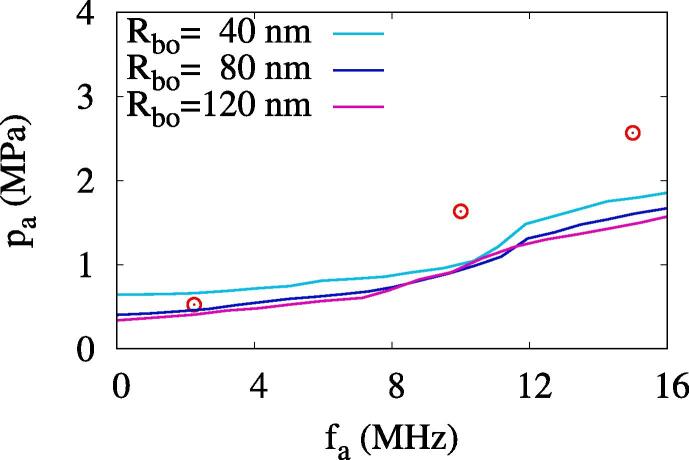
Effect of initial bubble radius on the ADV threshold with $R_{\mathrm{do}}=0.47\;\mu \text{m}$. The circle symbols represent the experimental data [16].

Fig. 9 presents the effect of $R_{\mathrm{bo}}$ on the ADV threshold. The ADV threshold pressure is observed to slightly increase as $R_{\mathrm{bo}}$ decreases. However, in the range of $40\mathrm{nm}⩽R_{\mathrm{bo}}⩽120\mathrm{nm}$, the ADV threshold does not change much, and the tendency of the threshold increase with frequency does not change. This means that the choice of $R_{\mathrm{bo}}=80\mathrm{nm}$ is not a very limiting condition for investigating the ADV threshold.

### 3.4 Effects of water properties and ambient temperature

Computations are performed to investigate the effects of water surface tension $\sigma_{\mathrm{dw}}$ and water viscosity $\mu_{w}$ on bubble growth in ADV. Such effects are implicitly included in the vaporization of droplets, which are typically phospholipid-coated or encapsulated in ADV applications. The encapsulation tends to reduce the surface tension and increase the viscosity effect due to the additional shell viscosity [35]. The analysis of Doinikov et al. [21] for ADV showed that the numerical predictions of bubble growth can be matched with the experimental data by varying the water surface tension and water viscosity to account for the encapsulation effect.

**Fig. 10:**
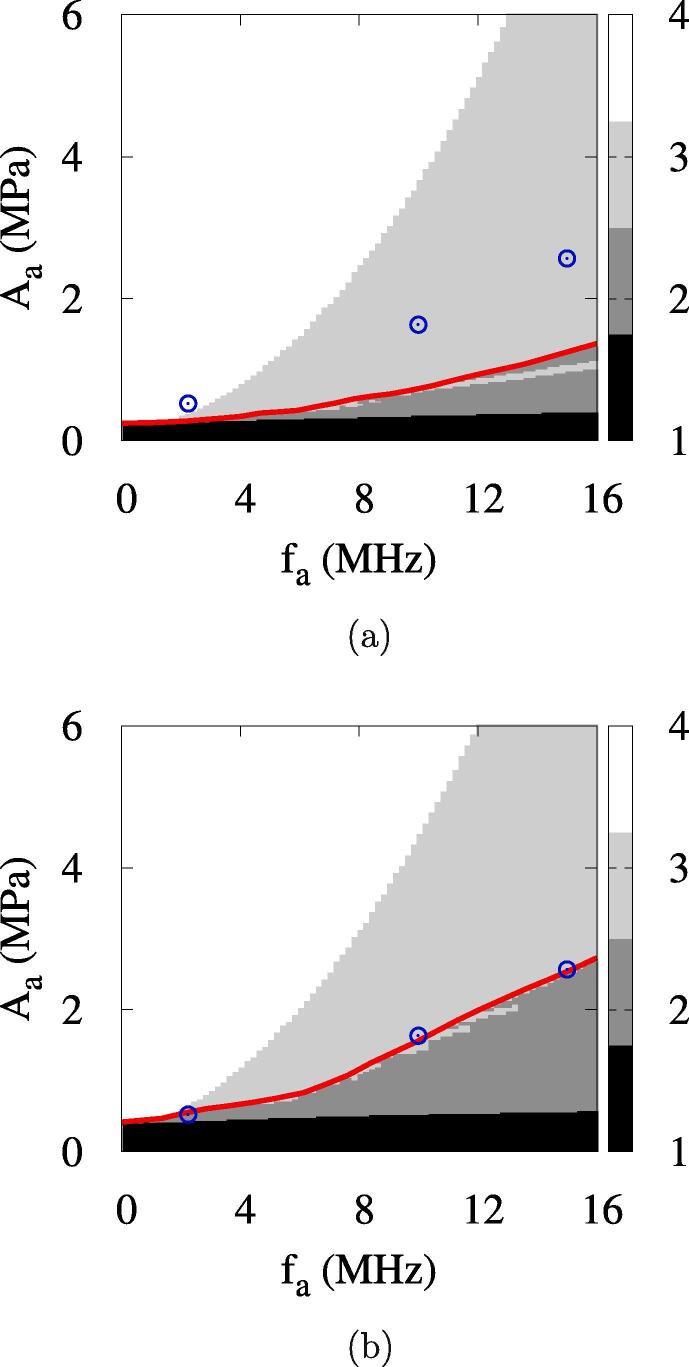
Influences of water surface tension $\sigma_{\mathrm{dw}}$ and water viscosity $\mu_{w}$ on the bubble growth regimes for ADV at $R_{\mathrm{do}}=0.47\;\mu \text{m}$: (a) $\sigma_{\mathrm{dw}}=1.25\times 10^{-2}N/m$ and (b) $\mu_{w}=4\times 10^{-3}\mathrm{Pas}$. In the figure, the red lines and blue circles represent the present numerical predictions of the ADV threshold and the experiment data [16]., respectively. (For interpretation of the references to colour in this figure legend, the reader is referred to the web version of this article.)

Fig. 10a presents the acoustic pressure-frequency diagram for bubble growth regimes when $\sigma_{\mathrm{dw}}$ is reduced by four times to $\sigma_{\mathrm{dw}}=1.25\times 10^{-2}N/m$. Assuming that a bubble nucleates at $T_{\mathrm{bo}}=T_{\infty }=310\;\text{K}$, its pressure is $p_{\mathrm{bo}}=0.131\;\text{MPa}$, as described in Section 2.2. The acoustic amplitude required for bubble nucleation is evaluated from Eq. (31) as $A_{a}>0.26\;\text{MPa}$, which is 38% lower than that for $\sigma_{\mathrm{dw}}=5\times 10^{-2}N/m$. The effective ADV threshold $A_{\mathrm{eth}}$ for $\sigma_{\mathrm{dw}}=1.25\times 10^{-2}N/m$ is observed to be reduced by about 30 %.

The ADV results at four times increased water viscosity to $\mu_{w}=4\times 10^{-3}\mathrm{Pas}$ are plotted in Fig. 10b. Compared to Fig. 7b for ADV at a lower water viscosity, the increased viscosity has a significant effect on $A_{\mathrm{eth}}$, but its effect is weak for regime 1 of irreversible bubble collapse without rebound. This is expected from the fact that the viscosity effect is remarkable in regimes with bubble oscillations of collapse and rebound. The effective ADV threshold $A_{\mathrm{eth}}$ for $\mu_{w}=4\times 10^{-3}\mathrm{Pas}$ increases by about 140 %.

**Fig. 11:**
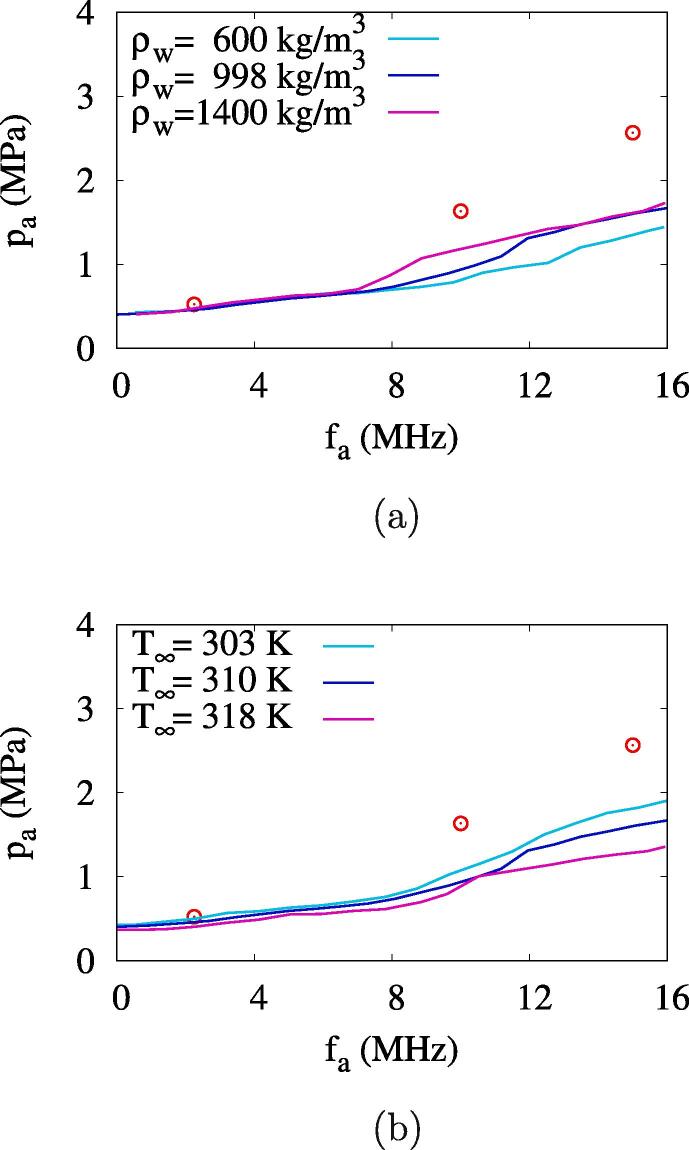
Influences of water density $\rho_{w}$ and ambient temperature $T_{\infty }$ on the ADV threshold with $R_{\mathrm{do}}=0.47\;\mu \text{m}$. The circle symbols represent the experimental data [16].

Fig. 11a shows the effect of water density $\rho_{w}$ on the effective ADV threshold $A_{\mathrm{eth}}$. When $f_{a}⩽7\;\text{MHz},A_{\mathrm{eth}}$ is not sensitive to $\rho_{w}$. However, in a higher frequency range, $A_{\mathrm{eth}}$ increases with $\rho_{w}$. This means that the energy required for the bubble to push the heavier surrounding liquid increases under higher frequency conditions with more bubble oscillations. The influence of ambient temperature $T_{\infty }$ on $A_{\mathrm{eth}}$ is depicted in Fig. 11b. As $T_{\infty }$ increases from 303K to 310K and 318K, the effective ADV threshold decreases by about 10 %, respectively. This indicates that $A_{\mathrm{eth}}$ decreases as the evaporation heat transfer increases with $T_{\infty }$.

### 3.5 Effect of initial droplet radius

**Fig. 12:**
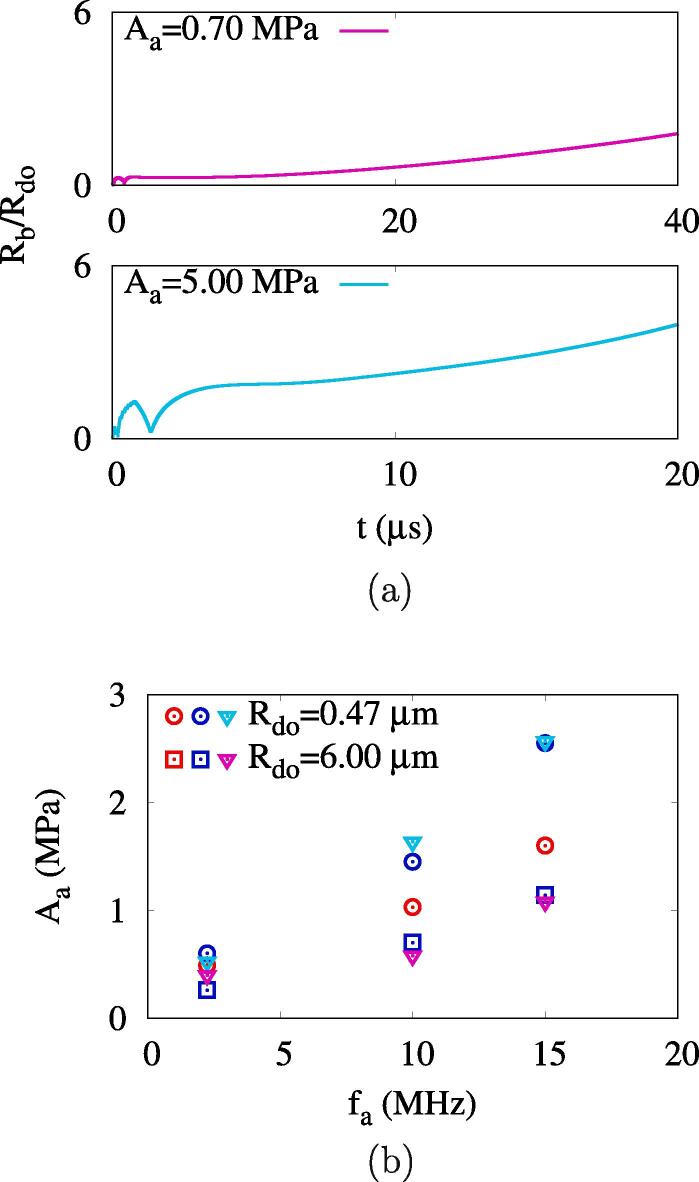
Effect of droplet radius on ADV: (a) the bubble growth at $R_{\mathrm{do}}=6\;\mu \text{m}$ and $f_{a}=10\;\text{MHz}$ and (b) the effective ADV threshold. In the figure b, the red, blue and triangle symbols represent the numerical predictions at $\mu_{w}=1\times 10^{-3}\mathrm{Pas}$ and $\mu_{w}=4\times 10^{-3}\mathrm{Pas}$ and the experimental data, respectively. (For interpretation of the references to colour in this figure legend, the reader is referred to the web version of this article.)

Fig. 12a shows bubble growth for ADV at the initial droplet radius increased to $R_{\mathrm{do}}=6\;\mu \text{m}$. As $R_{\mathrm{do}}$ increases, the period of bubble growth is not complete during the acoustic pulsing periods of $8/f_{a}$. After the pulsing period, the bubble is observed to grow slowly because of the evaporative heat transfer from the surrounding liquid.

The effects of initial droplet radius $R_{\mathrm{do}}$ and water viscosity $\mu_{w}$ on the effective ADV threshold $A_{\mathrm{eth}}$ are plotted in Fig. 12b. As the acoustic frequency $f_{a}$ increases, $A_{\mathrm{eth}}$ is observed to increase regardless of the magnitude of $R_{\mathrm{do}}$ and $\mu_{w}$. $A_{\mathrm{eth}}$ increases with increasing $\mu_{w}$ in the case of a small droplet, but it has little effect on $\mu_{w}$ for a large droplet of $R_{\mathrm{do}}=6\;\mu \text{m}$. When $R_{\mathrm{do}}$ increases from 0.47μm to 6μm, $A_{\mathrm{eth}}$ decreases, as observed in the experiment of Aliabouzer et al. [16]. The present predictions of $A_{\mathrm{eth}}$ are more comparable to the experimental data [16] at $R_{\mathrm{do}}=6\;\mu \text{m}$ than $R_{\mathrm{do}}=0.47\;\mu \text{m}$. The comparison is better when $\mu_{w}$ is increased by a factor of four.

## 4 Conclusions

A numerical model for the ADV threshold of microdroplets was developed by improving the Rayleigh-Plesset equation to properly treat the supercritical state occurring at rapid bubble collapse and by employing the van der Waals equation to consider the supercritical state. The assumption of bubble nucleation when the bubble nucleus is in thermal equilibrium with the surrounding liquid is observed to be reasonable for analysis of ADV. The numerical results showed four distinguished bubble growth regimes of irreversible collapse or complete vaporization with no rebound or multiple rebounds. The effective ADV threshold is observed to increase as the acoustic frequency increases and the initial droplet radius decreases, as experimentally observed in the previous studies. The ADV threshold increases with increasing the water viscosity in the case of a small droplet, but it has little effect on the water viscosity for a large droplet. The present numerical predictions of ADV threshold comparable to the experimental data.

## CRediT authorship contribution statement

**Sukwon Park:** Writing - original draft. **Gihun Son:** Writing - review & editing, Conceptualization, Methodology, Supervision.

## Declaration of Competing Interest

The authors declare that they have no known competing financial interests or personal relationships that could have appeared to influence the work reported in this paper.
